# Lights and Siren Transport and the Need for Hospital Intervention in Nontrauma Patients: A Prospective Study

**DOI:** 10.1155/2020/2651624

**Published:** 2020-03-16

**Authors:** Olivier Bertholet, Mathieu Pasquier, Elina Christes, Damien Wirths, Pierre-Nicolas Carron, Olivier Hugli, Fabrice Dami

**Affiliations:** ^1^Emergency Department, Lausanne University Hospital, University of Lausanne, CH-1011 Lausanne, Switzerland; ^2^Faculty of Medicine, University of Lausanne, Lausanne, Switzerland; ^3^Deputy Head in Charge of Community Policy, Lausanne City, CH-1000 Lausanne, Switzerland

## Abstract

**Background:**

The use of lights and siren transport (LST) has been a matter of debate because of the short time savings and well-established increased risks for Emergency Medical Services (EMS) and bystanders. Time-critical hospital intervention (TCHI) denotes urgently needed procedures that cannot be performed properly in an out-of-hospital setting. Since 2013, rapid transportation from the field, *fast-track*, is currently used for patients with acute ST-elevation myocardial infarction, suspicion of acute stroke and out-of-hospital cardiac arrest. The aim of this study was to determine whether the use of LST was associated with the realization of TCHI for nontrauma cases within 15 minutes of hospital arrival, to quantify overtriage (LST without TCHI) and to identify the predictors of TCHI.

**Methods:**

This is a monocentric prospective observational study of nontrauma patients transported by ambulance. Based on Ross et al.'s work in 2016 on trauma patients, TCHI procedures were developed by the study team. Descriptive statistics were used to determine whether the use of LST was associated with the realization of TCHI. Multivariable analyses determined the predictors of TCHI and compared clinical outcomes.

**Results:**

On the 324 patients included, 67 (20.7%) benefitted from LST, with 40 (59.7%) receiving TCHI (*p* < 0.001). The overtriage rate was 40.3%. The most common medical TCHI was the *fast-track* (65.2% of all TCHI). LST was predictive of the need for TCHI (*p* < 0.001), as was the clinical condition of the patient and also when EMS providers expected TCHI.

**Conclusions:**

A majority of the LST benefitted from TCHI with an overtriage rate of 40%. To reduce the rate of overtriage (LST without TCHI), LST should mainly be used for *fast-track* and when TCHI is expected by the EMS providers.

## 1. Background

The prehospital lights and siren transport (LST) of patients from the field to the hospital has been a matter of debate for several years. LST is perceived as a method for reducing travel time between the field and the hospital [1, 2]. On the other hand, LST is potentially associated with an increased risk of collision and injury to Emergency Medical Services (EMS) providers, patients, and bystanders [1–7]. Furthermore, the time saved, a couple of minutes, is considered moderate, with no clinical benefit demonstrated to date [8–11]. It is therefore important to reduce its use whenever possible.

There is currently no consensus on what would be an appropriate use of LST from the field to the hospital, and LST criteria for use are rarely defined in EMS protocols. A relatively simple way to assess LST adequacy has been to consider it appropriate when dealing with a potentially life-threatening situation [11–14]. Some studies have applied a more comprehensive approach, using the application of time-critical hospital intervention (TCHI) as an endpoint to indicate the appropriateness of LST [2, 9, 10, 15]. TCHI is defined as those procedures or treatments that are urgently needed, requiring skills or devices that are either not available or cannot be properly performed in the prehospital setting [2, 8, 10]. Recent studies have demonstrated that up to a quarter of life-threatening situations, such as airway obstruction, severe dyspnoea, hemodynamic instability, and abnormal Glasgow Coma Scale, require TCHI [8–10]. As proposed by the American Heart Association and the American Stroke Association since 2013, time-sensitive conditions (Mission: Lifeline®) such as acute ST-elevation myocardial infarction (STEMI), suspicion of acute stroke, and out-of-hospital cardiac arrest (OHCA) require rapid transportation from the field to benefit from *fast-track* procedure within the hospital (coronarography, thrombolysis, CT scan, and MRI) and therefore also may be considered as TCHI [16–18].

Most studies on TCHI were retrospective and centered on trauma cases. As non-trauma cases represent the majority of EMS case-mixes, based on a previous work on trauma patients [8], the present study aimed to assess prospectively the association between the use of LST and the rate of TCHI performed within the first 15 minutes of emergency department (ED) arrival on nontrauma cases and, therefore, quantify overtriage (LST without TCHI). Predictors associated with the realization of TCHI as well as patients' clinical outcomes were also collected.

## 2. Methods

This monocentric prospective observational study was conducted from October 2016 to April 2018 in the ED of Lausanne University Hospital in the State of Vaud, Western Switzerland (∼794,000 inhabitants in 2017). This Level-1 trauma centre has 1400 beds, and its ED provides 42,000 consultations per year. A unique medical dispatch centre coordinates the state's EMS crews. Paramedics use the state protocols for autonomous intravenous access, cardiopulmonary resuscitation procedures, defibrillation and emergency medication administration (acetylsalicylic acid, adrenaline, amiodarone, clonazepam, diazepam, fentanyl, glucagon, glucose, isosorbide dinitrate, midazolam, morphine, naloxone, paracetamol, salbutamol, and thiamine) [19]. The decision to use LST for transportation from the site is left to the discretion of the EMS crew [11] except when hospital physicians validate a *fast-track* procedure (STEMI, Stroke, and OHCA) which implies the use of LST. Its use allows the EMS crew to break normal traffic laws but with extreme caution. As patients arrive at the hospital, ED physicians do not know if the EMS crew used LST.

All patients arriving by ambulance at the ED were eligible. Trauma patients, hospital transfers, and patients under 16 years of age were excluded. As resources were not sufficient to follow-up all patients individually for the duration of the study, a convenience sampling method was used [20]. During their shifts, two research nurses and a medical student screened as many patients transported to the ED as possible. Inclusion was not possible 24/7.

As for trauma cases, TCHI procedures for nontrauma patients were not clearly defined or validated in the literature at the time of the study. The study team, therefore, adapted a list developed by Ross et al. [8] (Table 1).

The following prehospital variables were collected: gender; age; duration; and distance of transport from the field and the use of LST. Age was dichotomized (<65 vs. ≥65 years). In order to study those variables that may affect the delay and realization of TCHI, EMS providers were asked after each intervention if they expected TCHI to be performed. TCHI foreseen by the EMS providers was defined as “expected TCHI” and that performed was defined as “validated TCHI”. EMS providers estimated the severity of nontrauma cases using the National Advisory Committee for Aeronautics (NACA) score, which comprises eight categories ranging from 0 (no injury or disease) to 7 (lethal injuries or disease, with or without resuscitation attempts). A NACA score of ≥4 implies a potential life-threatening condition [21, 22].

The following hospital variables were collected: time interval between arrival in the ED and the first TCHI; TCHI performed within the first 15 minutes; inhospital length of stay (LOS); hospital mortality; and disposition after ED management in intensive care unit (ICU), intermediate care unit (IMCU), general ward (GW), surgery room, or ambulatory care. An arbitrary cutoff of 15 minutes for TCHI was chosen based on previous studies [8, 15].

Data were retrieved from the patient information database that was established for this study and analysed with the Statistical Package for the Social Sciences (SPSS), version 25 (IBM Corp., Armonk, NY, USA).

As appropriate, data were described as frequency, mean, and standard deviation (SD) or median and interquartile range (IQR). Descriptive statistics were used to analyse the frequency of LST use. Univariate analysis (including Student's *t*-test and Pearson's chi-square test) and multivariable analysis (including logistic regression) were used to determine variables associated with the receipt of TCHI: patients' age and gender; LST; NACA score; and expected TCHI. Odds ratio (OR), lower and upper confidence intervals (95%CI), sensitivity, specificity, and positive and negative predictive values (PPV and NPV) were calculated. A *p* value of <0.05 was considered to indicate statistical significance.

Use of LST without TCHI was considered as overtriage as risks are taken without benefit. However, TCHI without LST could not systematically be considered as undertriage as some situations do not automatically require LST (short transport distances to the hospital and/or fluid traffic conditions for example).

## 3. Results

A total of 324 patients were enrolled between October 2016 and April 2018. Thirteen patients were transferred from the ED to regional hospitals for continuation of care so their post-ED care and mortality data could not be collected. The mean age of the sample was 65 (SD 22) years, and 160 (49.4%) were male. Sixty-seven patients (20.7%) were transported with LST, 40 (59.7%) of whom benefitted from at least one TCHI (*p* < 0.001). Overtriage rate was 40.3%. Among the 257 (79.3%) patients transported without LST, 6 (2.3%) received TCHI. When transported with LST, patients had a NACA score of ≥4 (86.6% vs. 7.8%; *p* < 0.001). Median transport duration was 14 minutes without significant difference regardless of the use of LST; median transport distance was 13 kilometres (IQR 4.4–27.0) with LST comparing to 4.0 (IQR 2.7–7.0) without LST (*p* < 0.001). Mean time interval from arrival to the first TCHI was 7.7 (SD 4.0 minutes) with LST versus 9.3 (SD 6.4) minutes without LST (*p*=0.414) (Table 2).

The sensitivity and specificity of the transport mode to indicate the need for TCHI was 87.0% (95%CI = 77.3–96.7) and 90.3% (95%CI = 86.8–93.8), respectively. PPV was 59.7% (95%CI = 47.9–71.4), and NPV was 97.7% (95%CI = 95.9–99.5) (Table 3).

Less than 2% of TCHI was performed when EMS providers did not use LST and did not expect TCHI to be realized (NPV = 98.4%: 95%CI = 96.8–99.9). When EMS providers expected TCHI (*n* = 50), it was performed in 68% of cases (PPV = 68.0%, 95%CI = 55.1–80.9; NPV = 98.5%, 95%CI = 97.0–99.9; sensitivity = 89.5%, 95%CI = 80.6–98.4; and specificity = 94.4%, 95%CI = 91.7–97.1; *p* < 0.001). LST, NACA ≥4, and TCHI expected by EMS providers were the most predictive variables for the need for TCHI (*p* < 0.001) (Table 4).

The most common TCHI within 15 minutes of hospital arrival was *fast-track* for patients presenting with STEMI, stroke, or OHCA (*n* = 30), then intensive therapeutic medical procedures (*n* = 19), followed by invasive vascular procedures (*n* = 7) and, finally, respiratory support procedures (*n* = 5). No patient died within this interval. *Fast-track* patients represent 44.8% of the LST used and 75% of the TCHI performed.

When patients were transported with LST, they were hospitalized significantly more often in acute care units (ICU, IMCU) and less often in GW or discharged from the ED (ambulatory care) (Table 5).

Independent of the use of LST, the average LOS was 5 ± 7 days, and the hospital mortality rate was 3.4% (*n* = 11).

## 4. Discussion

Nontrauma patients represent the majority of EMS cases. It is, therefore, important to study this specific population when addressing the appropriate use of LST. The majority (60%) of nontrauma patients transported with LST benefitted from TCHI in this study vs. 23% for Ross et al. Regarding overtriage, defined as LST patients without TCHI, the rate in this work was significantly lower than in the trauma patient study (40% vs. 77%) [8]. This suggests better clinical decision-making from EMS professionals in this setting, but also confirms there is room for improvement to avoid unnecessary risks while driving with LST. As in Ross et al.'s study [8], there were less than 5% of patients who benefitted of TCHI but were transported without LST. These cannot simply be categorized as undertriage as traffic conditions and/or short transport distances from the field to the hospital may allow to run without LST, as it was the case for the 6 TCHI patients without LST. However, due to the small sample of patients, this hypothesis shall not be generalized. LST transports present a longer median travel distance. This finding might be explained as this hospital drains STEMI and Stroke *fast-tracks* from the whole state.

Significant predictors of TCHI included the use of LST, a NACA score of ≥4, and an EMS expectation of TCHI which emphasize the good quality of assessment by EMS crews. The latter could be included in further research to analyse the choice of EMS transport mode.

Based on these findings, LST overuse remained nonnegligible, with a PPV (LST with TCHI) of only 59.7%. This was similarly described in previous studies [8, 9, 12, 13] and confirms the need to define evidence-based protocol for guiding EMS transport practice. Regarding those results, LST for nontrauma patients should mainly be used for *fast-track* or when EMS providers expect TCHI, taking note of traffic conditions. If these criteria were applied in this study, LST could have been reduced from 20.7% to 16.3%. Improvement of LST use requires additional education of EMS providers on TCHI if other studies are to confirm these findings.

This study is subject to several limitations. First, it is a single-centre study with short prehospital transport distances and durations and with paramedics having a high level of autonomy. Also, the availability of ED staff may have an influence on the rate of TCHI performed within 15 minutes.

Secondly, a convenience sample was used. Only true random samples produce representative estimates for demographic variables in at least 95% of samples; random time-block or business hour samples differ systematically from the population, although in this specific dataset, the magnitude of the differences was not large. However, for many research projects, these differences may not be clinically significant, which makes these results and discussion admissible [20]. Thirdly, given the small sample size, the precision of these results may be low.

## 5. Conclusions

In this prospective study, the use of LST was significantly associated with TCHI realization. This was principally due to *fast-track* validated from the field by hospital physicians. To reduce overtriage (LST without TCHI), LST should mainly be used when a *fast-track* is activated or when TCHI is expected by EMS providers. This needs to be confirmed by further studies.

## Figures and Tables

**Table 1:** List of TCHI procedures to be performed within 15 minutes of arriving at hospital.

*1. Airway and/or respiratory support procedures*
Patient intubation
Mechanical ventilation
High-frequency jet ventilation
Tracheostomy
Cricothyroidotomy
Thoracocentesis
Chest tube placement
Noninvasive ventilation

*2. Invasive vascular procedures*
Central line
Arterial line
Dialysis catheter
Endovenous pacemaker
Embolization

*3. Intensive therapeutic medical procedures*
Cardiopulmonary resuscitation
Extracorporeal membrane oxygenation
Shock managment (rapid fluid administration, vasopressors)
Emergency medication (antihypertensives, vasodilatators, antiarrhythmics, and antiepileptics)
External pacing
Intoxication treatment-antidote administration
Active rewarming
Transfusion, frozen fresh plasma, factor VIIa, or prothrombin complex concentrate

*4. Fast-track*
STEMI^a^*fast-track*
Stroke *fast-track*
OHCA^b^*fast-track*

^a^STEMI: ST-elevation myocardial infarction; ^b^OHCA: out-of-hospital cardiac arrest.

**Table 2:** Patients characteristics.

	Total	LST^a^	No LST	*p*
Total, *n* (% of all patients)	324 (100)	67 (20.7)	257 (79.3)	
Male, *n* (%)	160 (49.4)	41 (61.2)	119 (46.3)	0.030
Age, mean (SD)	65 (22)	65 (18)	65 (23)	0.937
Age <65 years (%)	138 (42.6)	30 (44.8)	108 (42.0)	0.686
NACA^b^ score ≥ 4, *n* (%)	78 (24.1)	58 (86.6)	20 (7.8)	<0.001
Median transport distance, km (IQR)	4.6 (2.9–9.9)	13.0 (4.4–27.0)	4.0 (2.7–7.0)	<0.001
Median transport duration, min (IQR)	14.0 (9.0–20.0)	14.0 (8.0–23.0)	14.0 (9.0–20.0)	0.830
Expected TCHI^c^, *n* (%)	50 (15.4)	39 (58.2)	11 (4.3)	<0.001
Validated TCHI, *n* (% of expected TCHI)	34 (68.0)	32 (82.1)	2 (18.2)	<0.001
Performed TCHI, *n* (%)	46 (14.2)	40 (59.7)	6 (2.3)	<0.001
Mean time to 1st TCHI, min (SD)	7.9 (4.3)	7.7 (4.0)	9.3 (6.4)	0.414
Length of stay, mean (days) (SD)	5.3 (6.7)	5.6 (6.4)	5.2 (6.8)	0.648

^a^LST: lights and siren transport; ^b^NACA: National Advisory Committee for Aeronautics; ^c^TCHI: time-critical hospital intervention.

**Table 3:** Transport mode and the need for TCHI^a^.

	TCHI received		
Transport mode	Yes	No	Total patients
LST^b^	40	27	67
No LST	6	251	257
Total patients	46	278	324

Sensitivity: 40/46 (87.0%); specificity: 251/278 (90.3%); positive predictive value: 40/67 (59.7%); negative predictive value: 251/257 (97.7%). ^a^TCHI: time-critical hospital intervention; ^b^LST: lights and siren transport.

**Table 4:** Predictors of TCHI realization.

Variable	95% confidence interval			
	Odds ratio	Lower	Upper	*p*
LST^a^	61.975	24.078	159.524	<0.001
NACA^b^ score ≥4	28.263	12.290	64.994	<0.001
^c^TCHI expected by EMS providers	55.788	23.744	131.077	<0.001
Male	1.914	1.006	3.643	0.048
Age below 65 years	0.940	0.499	1.771	0.848

^a^LST: lights and siren transport; ^b^NACA: National Advisory Committee for Aeronautics; ^c^TCHI: time-critical hospital intervention.

**Table 5:** Hospitalization wards from the ED (except for 13 patients transferred to regional hospitals).

	Total	LST	No LST	*p*
Total, *n* (% of all patients)	311 (100)	64 (20.6)	247 (79.4)	
ICU, *n* (%)	15 (4.8)	13 (20.3)	2 (0.8)	<0.001
Surgery room, *n* (%)	7 (2.3)	1 (1.6)	6 (2.4)	0.677
IMCU^a^, *n* (%)	72 (23.2)	31 (48.4)	41 (16.6)	<0.001
GW^b^, *n* (%)	133 (42.8)	10 (15.6)	123 (49.8)	<0.001
Ambulatory care, *n* (%)	83 (26.7)	9 (14.1)	74 (30.0)	0.010

^a^IMCU: intermediate care unit; ^b^GW: general ward.

## Data Availability

Full collected data can be obtained through email from the corresponding author.

## Abbreviations

CI: Confidence interval
ECG: Electrocardiogram
ECMO: Extracorporeal membrane oxygenation
ED: Emergency department
eFAST: Extended focused assessment with sonography for trauma
EMS: Emergency medical services
GW: General ward
ICU: Intensive care unit
IMCU: Intermediate care unit
IQR: Interquartile range
LOS: Length of stay
LST: Lights and siren transport
NACA: National Advisory Committee for Aeronautics
NPV: Negative predictive value
OHCA: Out-of-hospital cardiac arrest
OR: Odds ratio
PPV: Positive predictive value
ROSC: Return of spontaneous circulation
SD: Standard deviation
SPSS: Statistical package for the social sciences
STEMI: ST-elevation myocardial infarction
TCHI: Time-critical hospital intervention.

## References

[1] Clawson J. (2002). Unnecessary lights-and-siren use: why are hundreds of people killed each year in collisions involving emergency vehicles?. *Public Management Review*.

[2] Tennyson J., Maranda L., Darnobid A. (2015). Knowledge and beliefs of EMS providers toward lights and Siren transportation. *Western Journal of Emergency Medicine*.

[3] Pirrallo R. G., Swor R. A. (1994). Characteristics of fatal ambulance crashes during emergency and non-emergency operation. *Prehospital and Disaster Medicine*.

[4] Studnek J. R., Bigham B. L., Martin-Gill C., Custalow C. B., Hawkins E. (2012). EMS provider and patient safety during response and transport: proceedings of an ambulance safety conference. *Prehospital Emergency Care*.

[5] Watanabe B. L., Patterson G. S., Kempema J. M., Magallanes O., Brown L. H. (2019). Is use of warning lights and sirens associated with increased risk of ambulance crashes? A contemporary analysis using National EMS Information System (NEMSIS) data. *Annals of Emergency Medicine*.

[6] Maguire B. J., Hunting K. L., Smith G. S., Levick N. R. (2002). Occupational fatalities in emergency medical services: a hidden crisis. *Annals of Emergency Medicine*.

[7] Custalow C. B., Gravitz C. S. (2004). Emergency medical vehicle collisions and potential for preventive intervention. *Prehospital Emergency Care*.

[8] Ross D. W., Caputo L. M., Salottolo K. M. (2016). Lights and Siren transport and the need for hospital intervention in trauma patients. *Prehospital Emergency Care*.

[9] O’Brien D. J., Price T. G., Adams P. (1999). The effectiveness of lights and siren use during ambulance transport by paramedics. *Prehospital Emergency Care*.

[10] Marques-Baptista A., Ohman-Strickland P., Baldino K. T., Prasto M., Merlin M. A. (2010). Utilization of warning lights and siren based on hospital time-critical interventions. *Prehospital and Disaster Medicine*.

[11] Dami F., Pasquier M., Carron P. N. (2014). Use of lights and siren: is there room for improvement?. *European Journal of Emergency Medicine*.

[12] Lacher M. E., Bausher J. C. (1997). Lights and Siren in pediatric 911 ambulance transports: are they being misused?. *Annals of Emergency Medicine*.

[13] Ismail A. K., Mohd Salleh N. I., Hamdan N. A. (2012). The use of warning lights and siren by the ambulance crew in the Universiti Kebangsaan Malaysia Medical Centre. *European Journal of Emergency Medicine*.

[14] Merlin M. A., Baldino K. T., Lehrfeld D. P. (2012). Use of a limited lights and siren protocol in the prehospital setting vs. standard usage. *The American Journal of Emergency Medicine*.

[15] Wydro G. C., Kruus L. K., Yeh E., Hatala K. M. (2007). Utilization of emergency lights and sirens by urban paramedics: analysis of indications for their use. *Annals of Emergency Medicine*.

[16] Granger C. B., Bates E. R., Jollis J. G. (2019). Improving care of STEMI in the United States 2008 to 2012. *Journal of the American Heart Association*.

[17] Powers W. J., Rabinstein A. A., Ackerson T. (2018). Guidelines for the early management of patients with acute ischemic stroke: a guideline for healthcare professionals from the American Heart Association/American Stroke Association. *Stroke*.

[18] McCarthy J. J., Carr B., Sasson C. (2018). Out-of-hospital cardiac arrest resuscitation systems of care: a scientific statement from the American Heart Association. *Circulation*.

[19] Public Health Service (2019). Paramedic’s algorithms. state of vaud (VD). https://www.vd.ch/fileadmin/user_upload/themes/sante/Professionnels/Mesures_sanitaires_d_urgence/Inter_soins/INTER_SOIN_ALGO_VD_SSP.pdf.

[20] Valley M. A., Heard K. J., Ginde A. A., Lezotte D. C., Lowenstein S. R. (2012). Observational studies of patients in the emergency department: a comparison of 4 sampling methods. *Annals of Emergency Medicine*.

[21] Raatiniemi L., Mikkelsen K., Fredriksen K., Wisborg T. (2013). Do pre-hospital anaesthesiologists reliably predict mortality using the NACA severity score? A retrospective cohort study. *Acta Anaesthesiologica Scandinavica*.

[22] Darioli V., Taffé P., Carron P. N. (2018). Evaluation of the discriminative performance of the pre-hospital National Advisory Committee for Aeronautics score regarding 48-h mortality. *European Journal of Emergency Medicine*.

